# Precision delivery of RAS-inhibiting siRNA to KRAS driven cancer via peptide-based nanoparticles

**DOI:** 10.18632/oncotarget.27109

**Published:** 2019-07-30

**Authors:** Matthew S. Strand, Bradley A. Krasnick, Hua Pan, Xiuli Zhang, Ye Bi, Candace Brooks, Christopher Wetzel, Narendra Sankpal, Timothy Fleming, S. Peter Goedegebuure, David G. DeNardo, William E. Gillanders, William G. Hawkins, Samuel A. Wickline, Ryan C. Fields

**Affiliations:** ^1^ Department of Surgery, Washington University School of Medicine, Saint Louis, MO, USA; ^2^ University of South Florida Health, Division of Cardiovascular Sciences, Tampa, FL, USA; ^3^ Norton Thoracic Institute, St. Joseph Hospital, Phoenix, AZ, USA; ^4^ Department of Medicine, Washington University School of Medicine, Saint Louis, MO, USA

**Keywords:** nanoparticle agents, pancreatic cancer, gastrointestinal cancer, oncoprotein

## Abstract

Over 95% of pancreatic adenocarcinomas (PDACs), as well as a large fraction of other tumor types, such as colorectal adenocarcinoma, are driven by KRAS activation. However, no direct RAS inhibitors exist for cancer therapy. Furthermore, the delivery of therapeutic agents of any kind to PDAC in particular has been hindered by the extensive desmoplasia and resultant drug delivery challenges that accompanies these tumors. Small interfering RNA (siRNA) is a promising modality for anti-neoplastic therapy due to its precision and wide range of potential therapeutic targets. Unfortunately, siRNA therapy is limited by low serum half-life, vulnerability to intracellular digestion, and transient therapeutic effect. We assessed the ability of a peptide based, oligonucleotide condensing, endosomolytic nanoparticle (NP) system to deliver siRNA to KRAS-driven cancers. We show that this peptide-based NP is avidly taken up by cancer cells *in vitro*, can deliver KRAS-specific siRNA, inhibit KRAS expression, and reduce cell viability. We further demonstrate that this system can deliver siRNA to the tumor microenvironment, reduce KRAS expression, and inhibit pancreatic cancer growth *in vivo*. In a spontaneous KPPC model of PDAC, this system effectively delivers siRNA to stroma-rich tumors. This model has the potential for translational relevance for patients with KRAS driven solid tumors.

## INTRODUCTION

Despite being one of the most prevalent and well-characterized proto-oncogene families, RAS proteins (HRAS, KRAS, NRAS) have largely eluded therapeutic intervention. Collectively, these small GTPases represent the most frequently mutated oncogene family in human cancer, present in up to 30% of all cases [1]. In particular, the KRAS proto-oncogene accounts for approximately 85% of all RAS mutations [2] and has been implicated in 95% of pancreas cancers [3] and 50% of colorectal cancers [4]. RAS encodes a 21-kD protein that cycles between an inactive, GDP-bound state, and an active, GTP-bound state. GTPase activating proteins (GAPs) are responsible for inactivating KRAS by hydrolyzing GTP, but oncogenic mutations of KRAS, most commonly at codon G12, confer resistance to inactivation by GAPs [5]. This leads to constitutive activation of KRAS and upregulation of downstream signaling cascades that promote many of the hallmarks of cancer, including sustained proliferation [6], metabolic reprogramming [7], resistance to apoptosis [8], immunological escape [9–11], cell migration [12], and metastasis [13, 14]. Mutant KRAS is well established as a true oncogenic driver – it is sufficient for neoplastic transformation *in vitro* [15], induces spontaneous tumor formation in genetically-engineered mouse models [16], and its expression is strictly required, even in advanced tumors [17]. Collectively, these features make KRAS one of the most attractive targets in cancer biology.

Indeed, in the 35 years since its discovery [18], KRAS has been the target of many attempts at pharmaceutical inhibition, including direct inhibition, interference with post-translational modification, disruption of membrane association, and interaction with downstream effectors [19]. However, no effective therapies targeting KRAS have entered the clinic, leading many to regard RAS oncoproteins as ‘undruggable’ [20]. Small-interfering RNA (siRNA) harbors tremendous therapeutic potential because it offers highly-specific, reversible control of gene expression [21]. A unique feature of siRNA therapy is the breadth of potential targets; essentially, any gene that is transcribed is a potential target. However, utilization of siRNA *in vivo* has been challenging due to a short circulating half-life, limited cellular uptake, and cellular confinement within endosomes [22, 23]. Prior studies looking at nanoparticles (NPs) to target KRAS and its associated pathway via siRNA have utilized various NP compositions, but unfortunately none of these have yet to make it to the clinic [24, 25]. Prior polymer and lipid based NP constructs are prone to cause generation of reactive oxygen species and calcium leakage, leading to off target effects, which is one potential advantage of our peptide based endosomolytic, oligonucleotide condensing NP [26–28]. In addition, the size of our NP (~55nm) and positive charge, unlike many prior NP formulations of various sizes and neutral or negative charge, enable us to target negatively charged tumor cells at the site of leaky tumor associated vasculature [29–32].

Prior work from our group has demonstrated that our peptide based p5RHH NP efficiently combines with siRNA, is taken up into tumor cells via micropinocytosis, and encapsulated in endosomes, whereby upon acidification of endosomes the NP is able to lyse the endosome membrane and deliver siRNA into the cytoplasm of the cell (peptide based, endosomolytic, oligonucleotide condensing nanoparticle) [26–28]. We hypothesized that this NP could deliver gene-level precision therapy to KRAS-driven tumors (Supplementary Figure 1). Herein, we employed this peptide-based nanocarrier, “p5RHH”, for the delivery of siRNA against KRAS, and assessed its propensity to: undergo cellular uptake, transmit siRNA, regulate gene expression, effect cellular viability, and alter tumor growth for KRAS-driven tumors.

**Figure 1:**
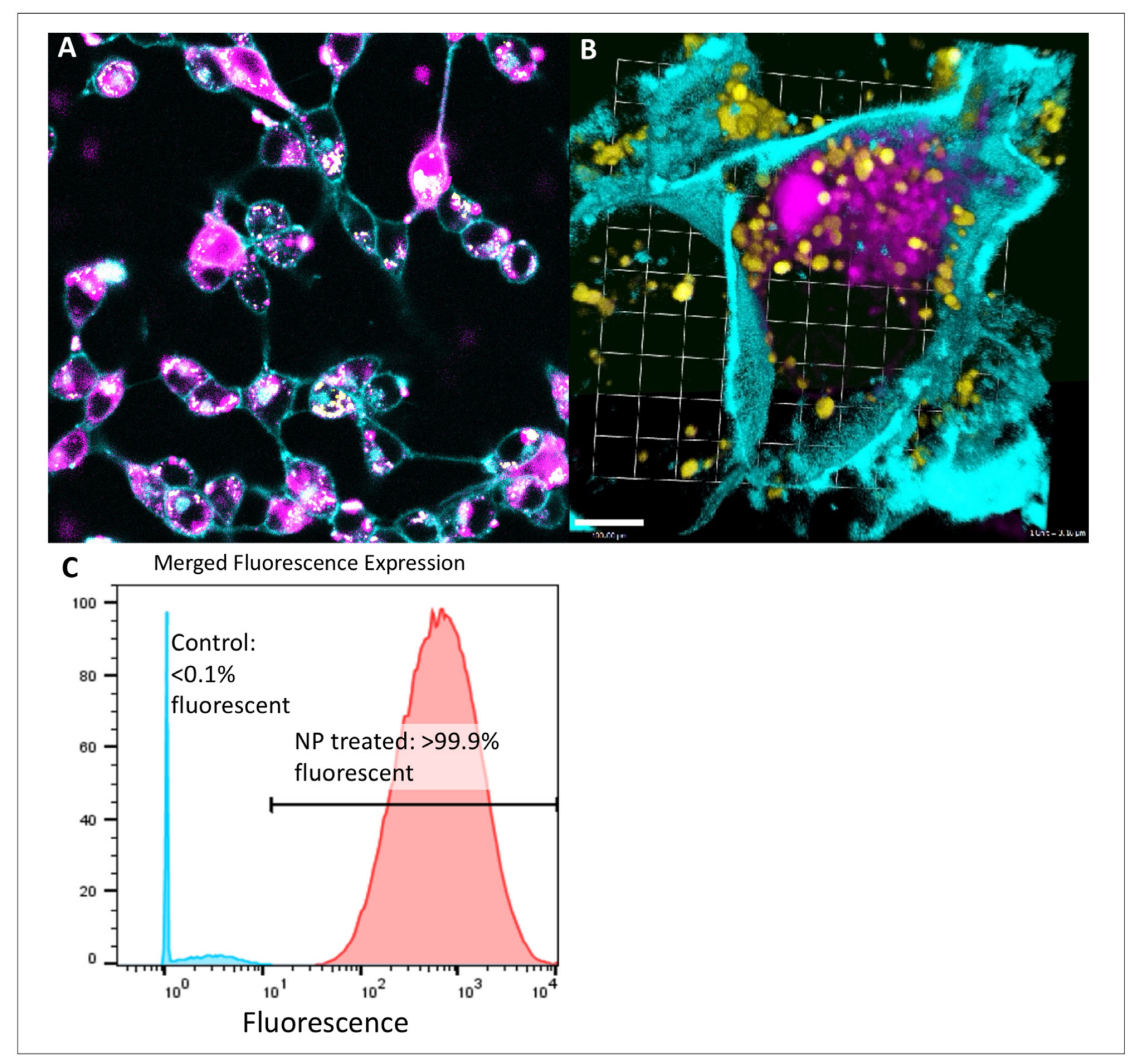
Intracytoplasmic delivery of siRNA by peptide nanoparticles in pancreatic and colorectal cancer is spatially separate from lysosomes and highly efficient. (**A**) Confocal microscopy demonstrates diffuse cell uptake of fluorescent tagged siRNA bearing NPs (pink) at 12 hours in CT26 cancer cells (cell wall cyan). (**B**) Confocal microscopy focusing on a single KPC-1 cancer cell (cell wall cyan) demonstrates accumulation of fluorescent signal (pink) in the cytoplasmic compartment, distinct from lysosomes (yellow), after administration of fluorescent siRNA-bearing peptide NPs. (**C**) Representative flow cytometry plot showing penetration of siRNA into the cytoplasm of KPC-1 pancreatic cancer.

## RESULTS

### Assessment of nanoparticle uptake *in vitro*

While this p5RHH oligonucleotide condensing, endosomolytic NP system has previously been used *in vivo* to successfully silence canonical NF-kB signaling in macrophages in models of rheumatoid arthritis and osteoarthritis [27, 28], we first wanted to assess the ability of this system to deliver siRNA into the cytoplasm of cancer cells *in vitro*.

Fluorescent NP were administered to pancreatic and colorectal cancer cells *in vitro,* and fluorescent and confocal microscopy were used to assess uptake. Using confocal microscopy, fluorescent cytoplasmic signal appeared to develop beginning 4 hours after administration of fluorescent NP. By 12 hours, the vast majority of cells appeared to contain fluorescent signal (Figure 1A). This strong signal continued at 24 hours time. Three-dimensional reconstruction images confirmed that fluorescent signal was present within the boundaries of the cell membrane, but was clearly distinct from lysosomes (Figure 1B).

Administration of fluorescent NP to cancer cells *in vitro* demonstrated a consistently high degree of uptake across 7 cell lines, as seen via flow cytometry (Table 1). The average percentage of cancer cells in a given line positive for fluorescent signal was 94.3%. A representative flow cytometry plot demonstrates >99.9% positivity for murine pancreatic cancer (Figure 1C).

**Table 1:** Nanoparticle uptake across multiple human and mouse pancreatic and colorectal cancers

Cell line	Cancer species	KRAS status	Mutation	Mutant alleles	% NP uptake
Pancreatic Ductal Adenocarcinoma					
BxPC-3	Human	WT	-	-	96.7
Capan-1	Human	MT	G12V	2	92.3
KCKO	Mouse	MT	G12D	1	83.5
KPC-1	Mouse	MT	G12A	1	99.6
Colorectal Adenocarcinoma					
CT26	Mouse	MT	G12D	2	99.9
MC38	Mouse	WT	-	-	93.8
WUC 322	Human	MT	G12D	1	94.0

NP = nanoparticle, WT = wild type, MT = mutant type.

### Assessment of KRAS pathway silencing *in vitro*

**Figure 2:**
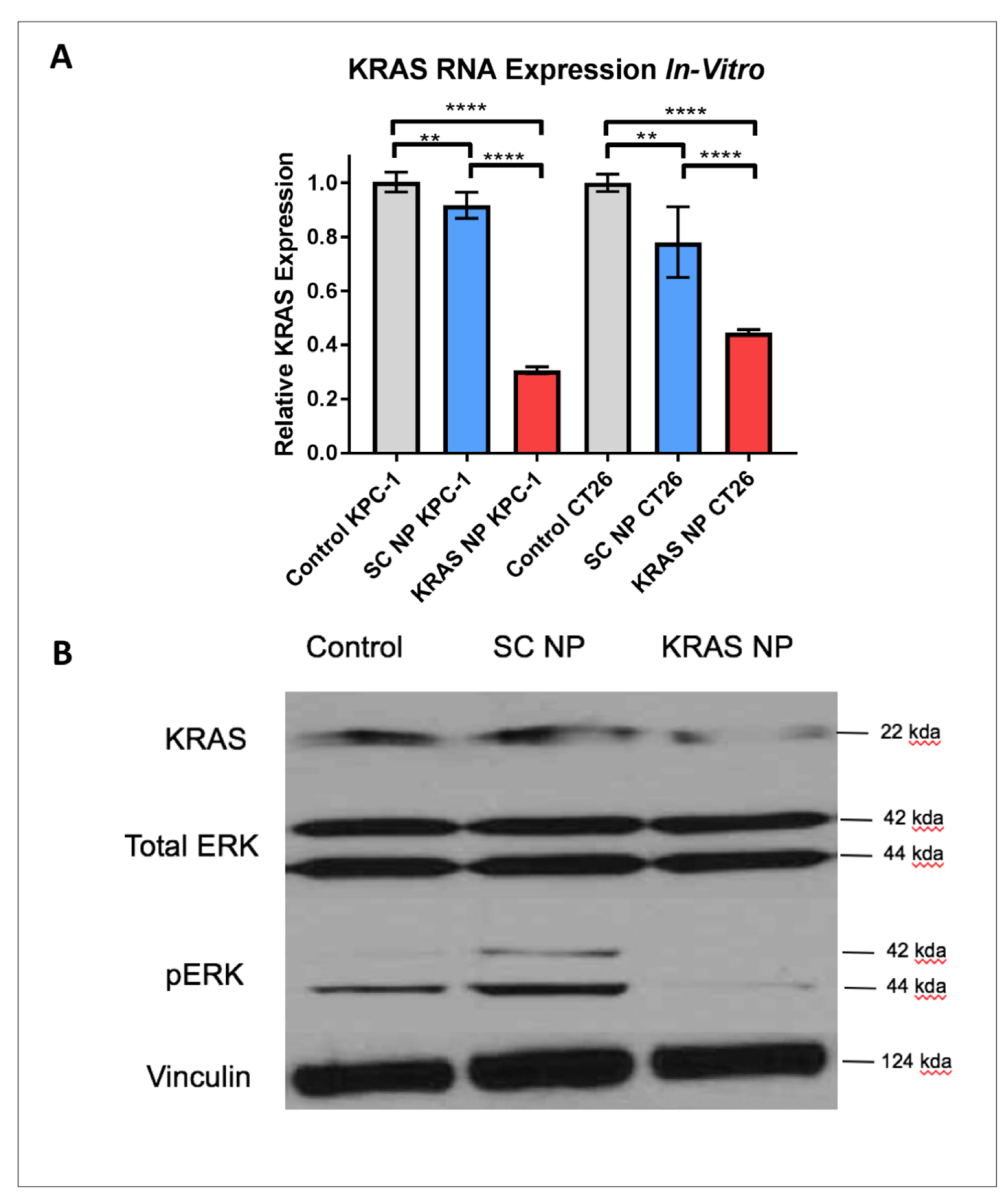
KRAS expression is reduced significantly in KPC-1 pancreatic cancer cells and CT26 colorectal cancer cells. (**A**) RT-PCR demonstrates decreased KRAS RNA expression in pancreatic (left side graph) and colorectal (right side graph) cancer cells treated with KRAS-siRNA NPs (KRAS NP), as compared to untreated cells (Control) or cells treated with scramble siRNA NP (SC NP). (**B**) CT26 colorectal cancer demonstrated decreased KRAS and phospho-ERK (pERK) expression after 24 hour treatment with KRAS NP treatment. ^****^=*p* < 0.0001, ^**^=*p* < 0.01, 95% confidence intervals shown.

After observing that every human and murine cancer cell line we tested took up fluorescent siRNA NP unequivocally and consistently, we next wanted to assess the efficacy of a silencing siRNA NP. Two separate KRAS driven murine tumor cell lines (KPC-1 and CT26) were treated with KRAS-siRNA NP *in vitro* for 24 hours. RNA was isolated from each group (3 replicates each) and RT-PCR was performed. At 24 hours, we observed a highly significant decrease in KRAS expression in those groups treated with KRAS-siRNA NP versus control cells or scramble (SC) siRNA NP treated cells (both *p < 0.0001*), with a reduction in KRAS RNA expression from 55–70% (Figure 2A). Western blotting demonstrated a qualitative reduction in the expression of KRAS and downstream pERK after KRAS-siRNA NP treatment for 24 hours (Figure 2B). This reduction of KRAS protein expression persisted 48 hours after administration (Supplementary Figure 2).

### Cell killing mechanism of KRAS silencing *in vitro*

**Figure 3:**
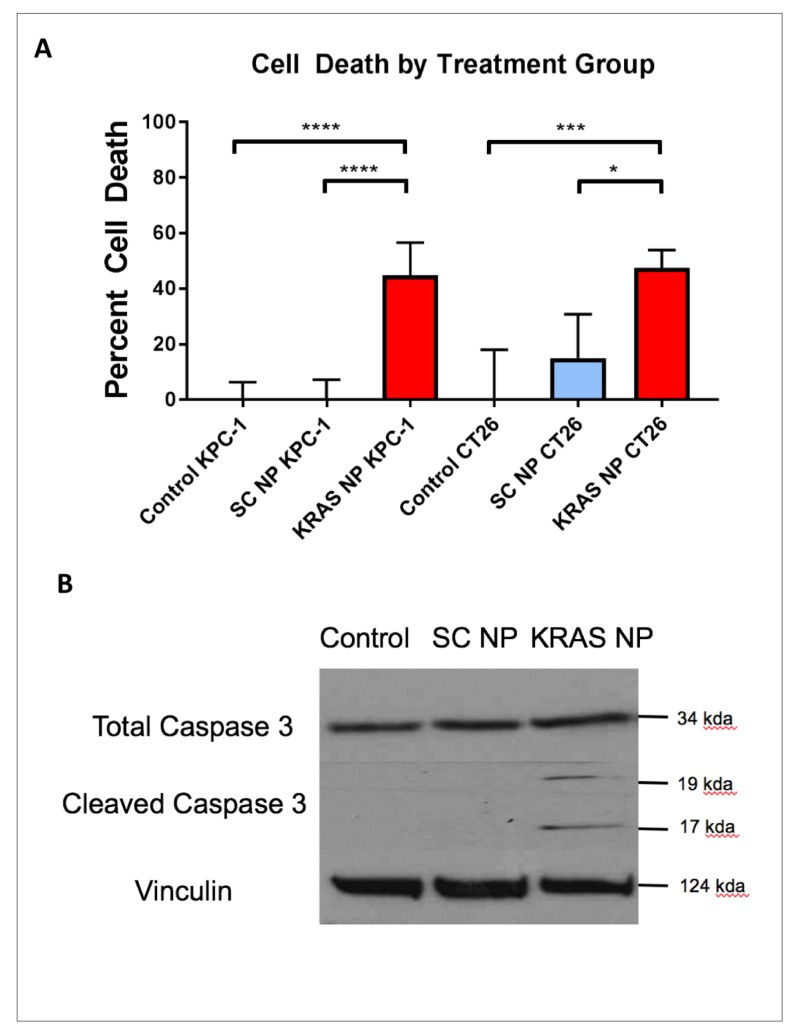
Treatment of pancreatic and colorectal cells with KRAS-siRNA NP leads to apoptosis mediated cell death. (**A**) KPC-1 pancreatic cancer cells (left) and CT26 colorectal cancer cells (right) demonstrate increased percent cell death after treatment with KRAS-siRNA NP. (**B**) CT26 colorectal cancer cells treated for 48 hours with KRAS-siRNA NP show upregulation of cleaved caspase 3. ^****^=*p* < 0.0001, ^***^=*p* < 0.001, 95% confidence intervals shown.

After observing that treatment with KRAS-siRNA NP *in vitro* reduced KRAS expression, we aimed to determine if KRAS knockdown in these cells could demonstrate any reduction in cell viability. KPC-1 and CT26 KRAS mutant cell lines were either left unexposed, or exposed to KRAS-siRNA NP or SC-siRNA NP for 48 hours. Cell death occurred in 53-55% of cells (both *p<0.001*) after treatment with KRAS-siRNA NP (Figure 3A). On multiple comparison analysis, KRAS-siRNA NP treatment resulted in a significant reduction in viability compared to both control (both *p<0.001*) and SC-siRNA NP treated cells (both *p<0.001*), while there was no significant difference in viability between control cells and SC-siRNA NP treated cells. In addition, at the same exposure time point, cleaved caspase 3, a marker of cellular apoptosis, was found to be upregulated in the KRAS-siRNA NP treated cells (Figure 3B).

### Assessment of nanoparticle uptake *in vivo*

**Figure 4:**
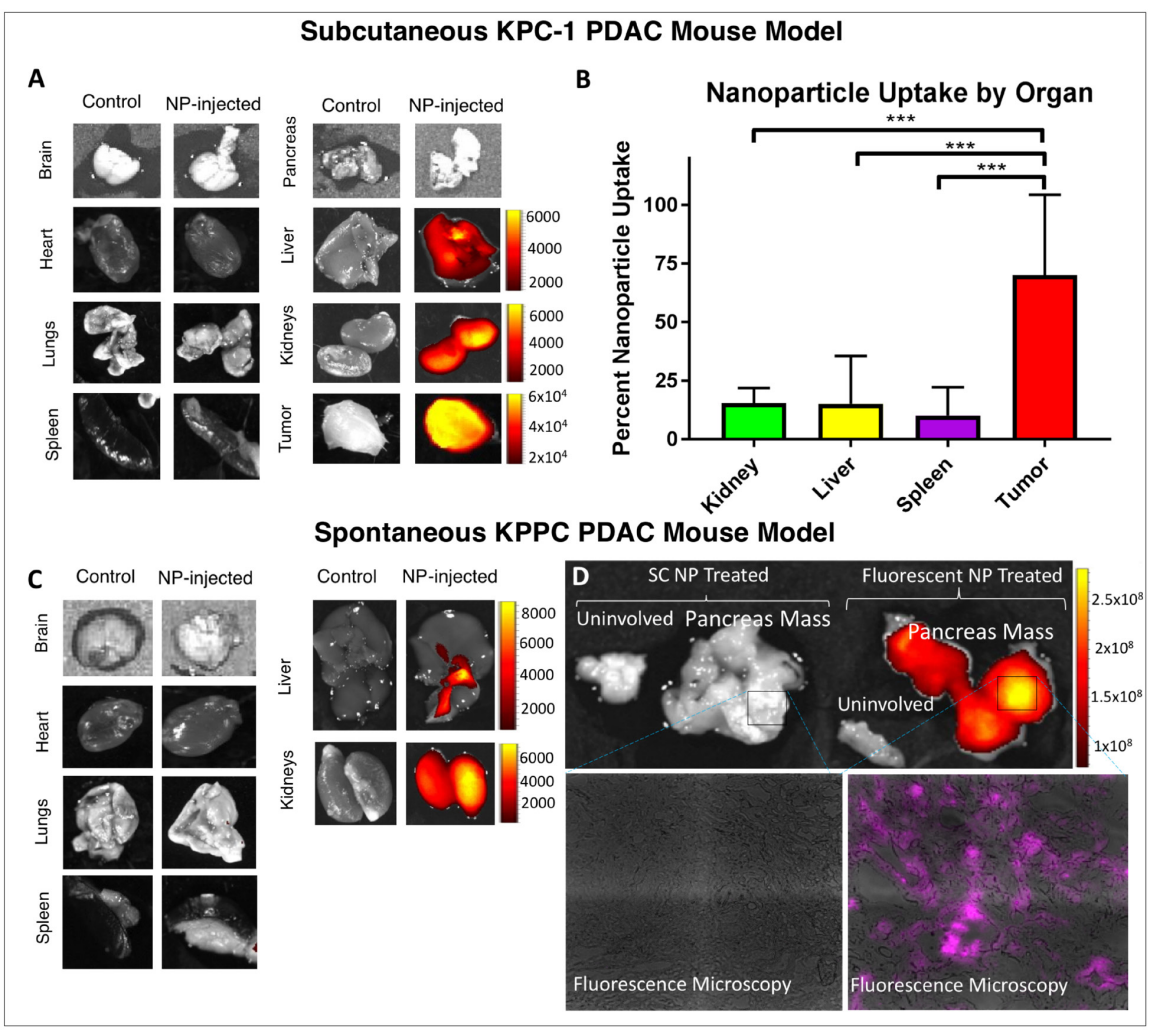
*In-vivo* tumor delivery of our nanoparticle construct. (**A**) The kidney, liver, and tumor of B6 mice growing KPC-1 tumors fluoresce after IV injection of fluorescent siRNA NP. (**B**) By flow cytometry, >70% of cells in the tumor are positive for fluorescent signal, while uptake in the kidney, liver and spleen is consistently < 20% ^***^indicates *p* < 0.001. (**C**) In KPPC mice (p48-CRE/Lox-stop-Lox(LSL)-Kras^G12D^/p53^flox/flox^), which develop spontaneous pancreas cancers, fluorescent signal is again seen in the liver and kidney using IVIS, consistent with excretion. (**D**) Fluorescent signal localizes to spontaneous pancreatic cancers with relative sparing of uninvolved pancreas, with IVIS results shown (top) and fluorescent microscopy (bottom). Quantification of IVIS in A, C, and D expressed as radiant efficiency ([photons/sec/cm^2^/steradian]/[W/cm^2^]). Please note scales showing order(s) of magnitude greater signal in tumors.

We next wanted to assess the *in vivo* distribution of intravenously-injected NPs to determine whether our formulation could penetrate the tumor microenvironment. We inoculated mice with the KPC-1 murine PDAC cell line to form subcutaneous syngeneic tumors, and injected (intravenous) these tumor-bearing mice with fluorescent siRNA-NP at specific tumor sizes, ranging from 2 mm to 2 cm. We evaluated tumor and organ fluorescence using an IVIS machine at serial time points post-injection. Results were consistent across all mice and tumor sizes: NP signal was evident in the tumor, liver, and kidneys. Signal was evident in the blood and all major organs within the first hour of injection. By six hours, signal was lost in the blood pool, but persisted in the tumor, liver, and kidneys. At 24 hours, the tumor, liver, and kidneys retained signal while the blood, brain, lungs, heart, pancreas, and spleen were negative (Figure 4A).

To further characterize the fluorescence imaging results from IVIS, we performed flow cytometry on single cell suspensions of tumors and tissues from tumor-bearing mice that received fluorescent NP injections. Twenty-four hours after administration of IV fluorescent NP, the liver, kidney, and spleen suspensions demonstrated fluorescence in an average of 15.1%, 15.4%, and 10.1% of cells, respectively, while the tumor demonstrated fluorescence in 70.1% of cells (Figure 4B). Representative flow cytometry plots are shown in Supplementary Figure 3.

Being convinced that our NP could deliver siRNA to the tumor microenvironment for tumors derived from cell lines, we sought to evaluate whether the system could be used to deliver NP to spontaneously arising tumors. For this we used mice with KRAS G12D and biallelic p53 mutations expressed in pancreatic tissue (KPPC mice), which leads to spontaneous PDAC tumor formation. KPPC mice were injected with fluorescent NP, and pancreata and the remaining organs were harvested at 24 hours post-injection. Fluorescent signal was detected in the liver and kidneys of these animals, but not in the brain, heart, lungs, or spleen. (Figure 4C). Assessment of the pancreas revealed strong fluorescent signal in the fluorescent siRNA-NP injected animal (nearly 5 orders of magnitude greater radiant efficiency as compared to liver and kidney IVIS signal); signal appeared most strong in areas of pancreatic mass, with sparing of uninvolved pancreatic tissue (Figure 4D). Fluorescence microscopy of the tumor confirmed siRNA delivery into the tumor microenvironment (Figure 4D). KPPC tumors have been shown to mirror the dense stromal infiltrate characteristic of human PDAC (Supplementary Figure 4) [33].

### Assessment of *in vivo* nanoparticle safety

To establish the safety of the NP platform, we assessed potential toxic effects of our NP treatment by conducting hematologic and biochemical studies on non-treated, and matched mice receiving SC- or KRAS-siRNA NPs. We focused these studies on renal and liver function, along with standard markers of inflammation and blood oxygen carrying capacity (Supplementary Figure 5). There was no difference among control, SC-siRNA NP, or KRAS-siRNA NP injected animals with respect to white blood cell count, hemoglobin, platelet count, aspartate aminotransferase (AST), alanine aminotransferase (ALT), creatinine, or sodium (Na). There was a statistically significant difference in blood urea nitrogen (BUN) concentration between control and KRAS-siRNA NP treated mice (29.5 vs, 22 mg/dl, *p<0.05*), however the clinical relevance of this difference is likely minimal, and if anything renal function appears to be enhanced in the KRAS-siRNA NP treated mice. Due to the use of CO2 euthanasia, serum potassium could not accurately be measured. The effect of our NP formulation on cardiac function was not assessed, as our p5RHH based NP has previously been shown to have no adverse effects on murine cardiac function in a model of atherosclerosis [34]. Finally, in studies looking at our p5RHH based NP in murine arthritis and atherosclerosis, after serial IV dosing, there was no activation of the innate or adaptive immune responses: namely, 1) no IgG or IgM to the intact peptide-siRNA NP or peptide itself; 2) no suppression of innate immune responsivity by splenocytes (anti-CD3–activated CD4^+^ T cells secreting normal levels of TNF-α, IFN-γ, IL-6, and IL-10); 3) no change in splenocyte subpopulations (CD4^+^ and CD8^+^ T cells, CD19^+^B cells, NK1.1^+^ natural killer cells, and Foxp3^+^ T regulatory cells); and 4) no activation of complement (C3a, 5a) [27, 28, 35].

### Assessment of KRAS pathway silencing *in vivo*

**Figure 5:**
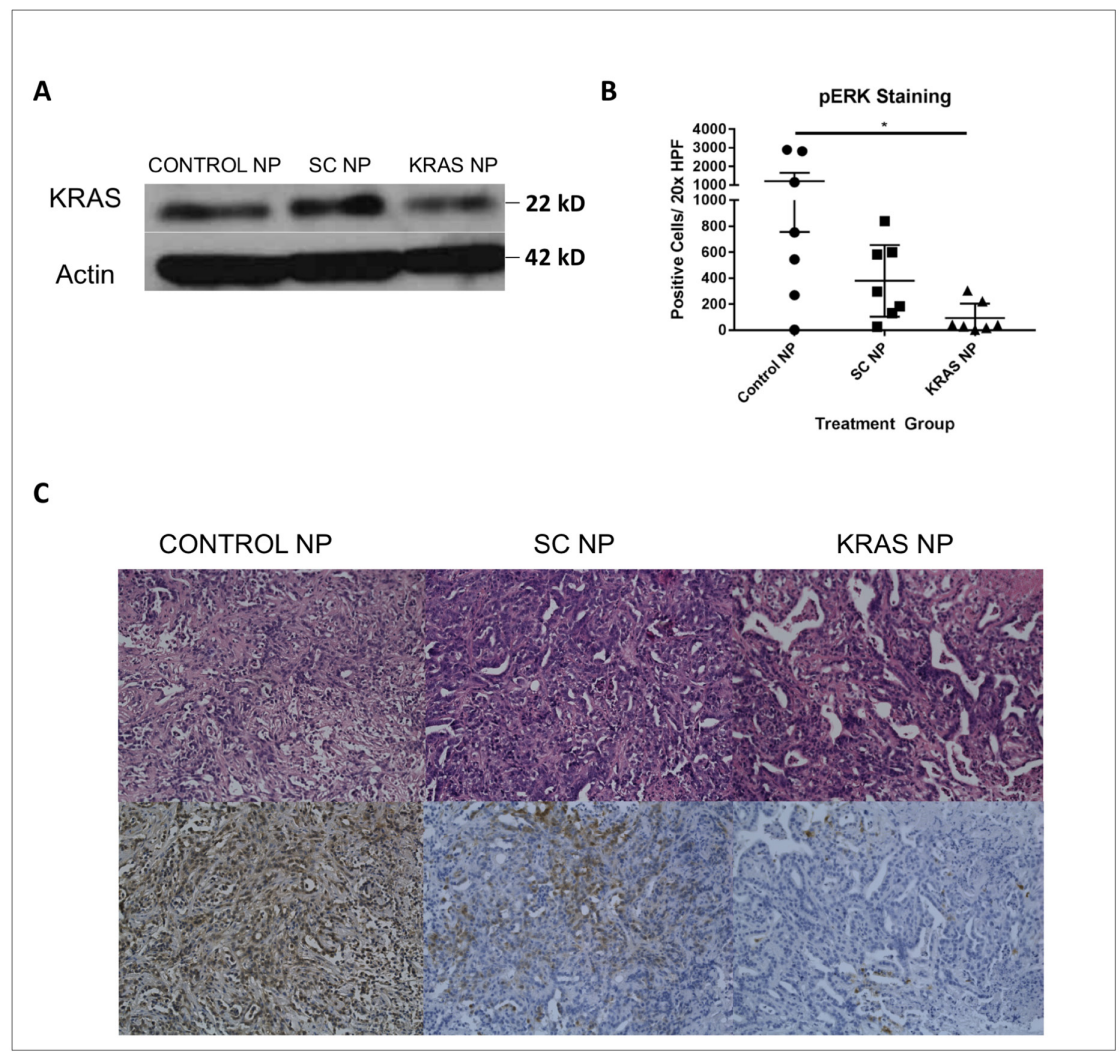
Tumors treated with KRAS siRNA NP demonstrate knockdown of the KRAS signaling pathway. (**A**) KPC-1 tumors from mice treated with KRAS-siRNA NP exhibit diminished KRAS protein level by Western blot analysis compared to scramble siRNA NP treated animals. (**B**) Looking at KPC-1 tumors from 7 mice/ group, with 5 20x images/ HPF taken, there was significant knockdown of pERK demonstrated in KRAS-siRNA NP (KRAS NP) treated mice. (**C**) H&E (top), and pERK IHC (bottom) images from control, scramble siRNA NP (SC NP) and KRAS NP treated mice. ^*^=*p* < 0.05, standard error of the mean (SEM) shown.

Having established that NP injection could reach the tumor microenvironment and appears to be safe under these conditions, we next sought to determine if delivery of a functionally-active siRNA could induce silencing of gene expression. KPC-1 tumor-bearing mice injected with anti-KRAS siRNA NP showed decreased tumoral KRAS protein expression as compared to SC-siRNA NP treated mice (Figure 5A). In addition, we demonstrated a robust, statistically significant, pERK knockdown after anti-KRAS NP treatment (*p<0.05*, Figure 5B and 5C, and Supplementary Table 1).

### Physiologic effect of KRAS silencing *in vivo*

**Figure 6:**
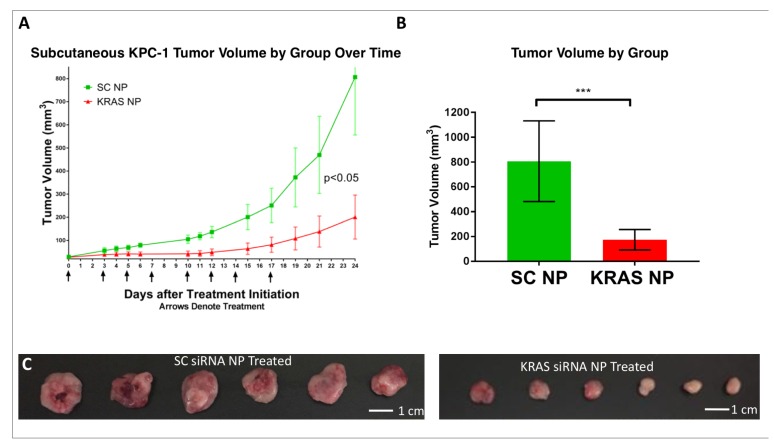
Demonstration of KPC-1 pancreatic cancer growth suppression *in-vivo.* (**A**) Treatment of KPC-1 tumor bearing mice, starting one week post tumor engraftment, with KRAS siRNA NP leads to a reduction in tumor growth rate versus that seen with scramble siRNA NP (SC NP) treatment. (**B**) Tumor volume is reduced 4.6 fold in mice receiving KRAS siRNA NP compared to mice receiving SC NP at 24 days. (**C**) Side by side *ex-vivo* images of KRAS-siRNA NP treated and scramble siRNA NP treated tumors. ^***^=*p* < 0.001, ^*^=*p* < 0.05, 95% confidence intervals shown.

To test the ability of an anti-KRAS NP to inhibit tumor growth, we inoculated mice subcutaneously with KPC-1 pancreatic cancer cells (2x10^5^ cells per tumor injection). Seven days after inoculation, mice were randomized to receive either SC-siRNA NP or KRAS-siRNA NP via tail vein injection. Five days after the initiation of treatment (2 treatments), there was a statistically significant difference in tumor volume between the two groups (tumor size assessed three times weekly by caliper assessment), which was maintained for the duration of the 3.5 week experiment (Figure 6A). The experiment was concluded at a point when any mouse developed ulceration of a tumor. Tumor volume at the conclusion of the experiment was suppressed in the KRAS siRNA NP group (*p<0.01*, Figure 6B). Representative images of the tumors at necropsy are seen in Figure 6C.

## DISCUSSION

Unfortunately, PDAC harbors a 5-year survival of only 8% [36]. Even with the most efficacious chemotherapy regimen, consisting of Fluorouracil, Leucovirin, Irinotecan, and Oxaliplatin (FOLFIRINOX), less than a third of patients with metastatic PDAC experience an objective response at 2 years follow-up, and median survival remains less than 1 year [37]. This large gap in treatment has led to a vast amount of research striving to improve survival in these patients; however, only a handful of clinical trials have yielded positive results [38]. These trials have invariably utilized standard, non-specific, traditional cytotoxic chemotherapeutic formulations and though survival has improved, it has been only by a few months.

Multiple studies have consistently demonstrated the critical role of KRAS in the overwhelming majority of pancreatic cancers and many colorectal cancers [17, 39]. A wide range of approaches to inhibiting mutant RAS have been explored in the three decades following its discovery as an oncogene, including: direct inhibition, interference with membrane association, inhibition of downstream effectors, and the exploitation of synthetic lethal interactors [19]. Despite this, no RAS inhibitor has reached the clinical arena. Direct targeting of the KRAS protein has been particularly challenging because the most common KRAS mutation consists of a single amino acid substitution, which has been inaccessible to stearic inhibitors thus far [40].

RNAi may represent a strategy to target KRAS while avoiding the challenge of inhibiting the protein directly. siRNA therapy is highly specific at the cellular level, and thus has the potential to have a favorable side effect profile, especially compared to cytotoxic chemotherapy. This specificity is supported by early clinical trials utilizing siRNA, in which it appears to be safe and well-tolerated [41, 42]. While highly specific at the cellular level, tissue-type specificity, such as to solid tumors, has been a major barrier to siRNA therapy. Additionally, the dense stroma known to accompany PDAC further complicates delivery and has hampered multiple previous therapeutic attempts [43].

Despite these significant hurdles, there has been some recent success in using nanoparticle based therapeutics coupled with RNA interference, such as a paper by Pei et. al., whereby they demonstrate sequential targeting of TGF-β followed by KRAS, which led to tumor uptake and decreased KRAS expression in murine models of PDAC [44]. Other groups have also had some early success targeting KRAS in PDAC and CRC tumor models [44–49]. Delivery to stroma rich PDAC remains the greatest challenge, and exciting novel strategies such as using superparamagnetic nanoparticles coupled with siRNA have also effectively targeted PDAC cells [47]. Other studies have utilized multiple non-KRAS siRNA targets to lead to therapeutic efficacy, such as one recent paper by Taniuchi and colleagues, whereby nanoparticles coupled to siRNA against six targets decreased prevalence of metastasis *in vivo* using murine PDAC models [48].

In this study, we show that a serum-stable, cell-penetrating, oligonucleotide-condensing, endosomolytic peptide-based NP system can deliver siRNA against KRAS to KRAS-driven cancers, reducing KRAS pathway expression, and slowing KRAS-driven pancreatic cancer growth *in vivo*. We demonstrate delivery of siRNA to the tumor microenvironment in the PDAC model KPPC, which recapitulates the dense stromal architecture of human PDAC and closely mimics the natural history of human pancreatic cancer [33, 50]. Our research team’s peptide-based p5RHH oligonucleotide-condensing NP has prior to this only been utilized *in vivo* for cardiovascular and arthritis applications [27, 28, 35].

Penetrating the stroma-dense tumor microenvironment has been an ongoing challenge in treating PDAC. Our NP system exploits the enhanced permeability and retention (EPR) effect that is known to accompany tumor vasculature. Though targeting moieties specific for PDAC have been used with some prior NP formulations, successful delivery from the vasculature into the tumor microenvironment still relies on NP influx from permeable tumor-associated vasculature [25]. Thus, NP size, charge, shape and geometry are crucial. Prior data looking at optimal sizing of NPs has established the “goldilocks” size of 50 nm (versus 20 or 200) being ideal for both ideal tumor microenvironment uptake and slow clearance [31]. In addition, much debate has occurred with regard to ideal NP charge, with no clear consensus of negative, neutral or positive NPs being more ideal [32]. Our NP construct is sized optimally at 55 nm, consistent with prior literature showing an ideal size of ~50 nm, with sizing situated to permeate leaky vasculature, stay at the tumor microenvironment, but not readily penetrate normal vascular barriers [26]. Our particle also exploits differences in cellular charge, that we feel is optimal for tumor drug delivery. While most mammalian cells maintain a charge-neutral or slight net positive charge via ion pumps, cancer cells typically harbor a net negative charge due to increased glycolysis and lactate secretion [26, 30]. Our p5RHH peptide NP system utilizes a positive charge (+12 mV) to promote preferential attraction to cancer cells, which differs from the majority of NP formulations used in the past, which tend to harbor a negative or neutral charge [26, 51].

In addition to the noted potential advantages in drug delivery, prior polymer and lipid based NP constructs are prone to cause generation of reactive oxygen species and calcium leakage, leading to off target effects [26–28, 52]. Our peptide based construct is predominantly cleared renally and has been found to have minimal off target effects both based on our data and our group’s prior work [26, 27, 29, 34]. One issue with prior peptide NPs had been endosomal uptake and sequestration, but our melittin derived p5RHH NP construct is by design endosomolytic and rapidly delivers siRNA to the cytoplasm of the target cell (Figure 1) [26]. Finally, although there may be concern regarding off targeting effect of our siRNA targeting KRAS, we demonstrated no significant toxicity in our murine model, with our KRAS siRNA or scramble siRNA NP (Supplementary Figure 5). This speaks to the lack of off target delivery of our p5RHH oligonucleotide condensing, endosomolytic NP construct (Figure 4).

We do acknowledge that there is variability seen in degree of KRAS knockdown across treatment groups in our *in vivo* experiments (Figure 5). However, the overall trend is clear, that KRAS signaling is suppressed after treatment with our KRAS-siRNA NP. Multiple factors such as timing of the last treatment dose and the timing of tumor takedown influenced data variability in our *in vivo* experiments, and although we strived to keep these variables as consistent as possible across groups, small variations were inevitable. In addition, although the small size and the positive charge of our NP make it uniquely capable of targeting cancer cells both *in vitro* and *in vivo*, we are working on incorporating targeting moieties into this NP system to further enhance specificity to tumor cells.

In summary, we demonstrate proof-of-concept that a serum-stable, cell-penetrating, and endosomolytic peptide-based NP system can deliver siRNA against KRAS both *in vitro* and *in vivo,* including in a spontaneously-arising KPPC PDAC mouse model. This led to reduced KRAS expression, resulting in apoptosis of KRAS driven tumors, and inhibition of KRAS-driven PDAC growth *in vivo*. These results are significant because they show that NP systems can be used to preferentially target tumor tissue and robustly deliver siRNA, enabling precise gene-level control within a stroma-dense tumor microenvironment. While our system has been effective in limiting RAS expression, this system could theoretically be used to target other drivers of tumor progression.

## MATERIALS AND METHODS

### Cell lines and maintenance

All cell lines were incubated in 5% CO2/95% air at 37°C. KPC-1 murine PDAC cells, which were initially derived from KPPC mice (p48-CRE/Lox-stop-Lox(LSL)-Kras^G12D^/p53^flox/flox^) spontaneous PDAC tumors, were a generous gift from the laboratory of Dr. David DeNardo [53]. MC38 colorectal cancer cells were a generous gift of Dr. David Linehan. WUC 322 is an in-house derived cell line from a mutant type KRAS metastatic colorectal cancer patient. Additional cells lines BxPC3, CT26, KCKO, and Capan-1 were obtained from the American Type Culture Collection (ATCC). KPC-1 cell line was cultured with DMEM:F12 50/50 mixture (Gibco) with 10% FBS, 2.5% HEPES buffer, and 1% antibiotic-antimycotic (Gibco). CT26 cell lines were cultured in RPMI with 10% FBS, 2.5% HEPES buffer and 1% antibiotic-antimycotic. The remaining cell lines were cultured in DMEM with 10% FBS, 2.5% HEPES buffer, and 1% antibiotic-antimycotic. KPC-1 line was maintained in culture on collagen-coated plastic, while all others were cultured without collagen. Cell lines were tested to confirm the absence of *Mycoplasma*. Cells for both *in vitro* and *in vivo* experiments were harvested using 0.05% trypsin at approximately 75-85% confluence.

### Nanoparticle preparation

The oligonucleotide-condensing peptide p5RHH [26] was synthesized by solid phase methods at GenScript (Piscataway, NJ, USA). For *in vitro* studies, NPs were generated by combining 10 mcL of 20 mcM siRNA with 1 mcL of 20 mM p5RHH in an RNAase-free microcentrifuge tube with 389 mcL of Optimem media (Life Technologies, Carlsbad, CA, USA) to produce a 400 mcL solution. The mixture was incubated for 40 minutes at 37° C. During incubation, standard culture media was removed and replaced with 900 mcL Optimem. After incubation, 100 mcL of solution was added to each experimental well of a 6-well plate. This was scaled as appropriate for different sized wells.

For *in vivo* studies, 10 μL (100 μM) siRNA was combined with 5 μL (20mM) p5RHH in 185 mcL Hank’s Buffered Salt Solution (HBSS) with calcium and magnesium (Life Technologies, Carlsbad, CA, USA). The mixture was incubated on ice for 6 minutes and 30 seconds. A 0.3 mL insulin syringe (Terumo) was used to administer 150 mcL of the NP solution via tail vein injection. These *in vitro* and *in vivo* protocols and have been reported previously [29].

### siRNA

Fluorescent tagged siRNA (Quasar705) were obtained from Sigma-Aldrich. Control siRNA (proprietary sequence) was obtained from GE Dharmacon. Targeted siRNA sequences purported to silence KRAS were obtained from Sigma-Aldrich and GE Dharmacon. siRNA was resuspended according to manufacturer’s instructions and RNA concentration was quantified using a NanoDrop microvolume spectrophotometer. siRNA concentrations were normalized and stored per manufacturer instructions. Among siRNA obtained from Sigma-Aldrich and GE Dharmacon, Sigma-Aldrich KRAS siRNA sequence GUGCAAUGAGGGACCAGUA (5’-3’) and its complementary antisense strand UACUGGUCCCUCAUUGCAC (5’-3’) were found to be the most efficacious in reducing murine KRAS mutant cancer cell viability (CellTiterGlo Assay) via traditional transfection methods and within our NP construct and was therefore selected for subsequent experiments (Supplementary Figure 6).

### Confocal microscopy

NP containing a Quasar705 fluorochrome tagged siRNA was used. Cells were observed by confocal fluorescent microscopy *in vitro* throughout treatment. Confocal microscopy was performed using a Zeiss LSM 880 confocal microscope. Cell membrane staining was performed using CellMask^TM^ Orange (Life Technologies, Carlsbad, CA, USA) per manufacturer instructions. Lysosomal staining was performed using LysoTracker^TM^ Green DND-26 (Life Technologies, Carlsbad, CA, USA) per manufacturer instructions.

### Cell viability assay

Cell viability assays were performed using the CellTiterGlo® luminescent viability assay, which quantifies the amount of ATP present at the time of cell lysis. Cells were seeded into a 96-well plate with 90 mcL of media, allowed to settle for 24 hours, and then received either no treatment (addition of 10 mcL media), treatment with scramble siRNA NP (SC-siRNA NP, 10 mcL), or treatment with KRAS-siRNA NP (10 mcL). Treatment lasted 48 hours, at which point the CellTiterGlo® assay was employed to determine cell viability. One hundred microliters of CellTiterGlo® solution was added to each well. Plates were gently agitated on an orbital shaker for 10 minutes, and luminescence was detected using a Biotek® Synergy HT plate reader at 450 nm.

### Animals and *in vivo* models

Male and female C57BL/6 and NOD-SCID (Prkdc^scid^) mice, age 8 to 12 weeks, were obtained from the Jackson Laboratory and cared for in a barrier facility under guidelines established by the American Association for Accreditation of Laboratory Animal Care as well as the U.S. Public Health Service policy on Human Care and Use of Laboratory Animals. The Washington University School of Medicine Institutional Animal Studies Committee approved all pertinent studies. Prior to injection into mice, cells were washed with PBS, and resuspended in 50 mcL of a 50/50 mixture of PBS and Matrigel (Corning). For KPC-1 subcutaneous cancer models, C57BL/6 mice were anesthetized with ketamine/xylazine, and after loss of pain reflex, placed in the lateral position, shaved, and injected with 2 x 10^5^ cells into the right flank using a 30 Ga syringe (Terumo). KPPC mice (p48-CRE/Lox-stop-Lox(LSL)-Kras^G12D^/p53^flox/flox^) used in these studies have been previously described [54] and were backcrossed to C57BL/6 background and screened for C57BL/6 identity using congenic markers.

For NP pharmacokinetic studies, mice were inoculated with cells as above. Once tumors reached at least 5 mm in greatest dimension, a single 150 mcL injection of Q705 fluorescent-siRNA NP was administered via tail vein injection. Twenty-four hours after injection, mice were sacrificed, and organs were imaged *ex vivo* using a Xenogen *In Vivo* Imaging System (IVIS).

For the subcutaneous KPC-1 pancreatic cancer model, mice were randomly assigned to receive no treatment, injection with SC-siRNA NP, or injection with KRAS-siRNA NP. Injections began day 7 after tumor inoculation, and were administered three times weekly for 8 total treatments. For KRAS protein knockdown experiments, injections occurred three times weekly for 1 week, followed by the mice being euthanized 24 hours after the 3^rd^ treatment dose. Tumor dimensions were measured to the hundredth of a millimeter three times weekly with calipers, and tumor volumes were calculated using the formula: volume = (L x W x W)/2, whereby L represents the greatest dimension of the tumor, and W is the measurement perpendicular to L [55]. Mice with spontaneous tumor regression were eliminated from analysis. Spontaneous tumor regression was defined as any tumor that fulfilled both criteria: 1) negative growth rate on three consecutive measurements, and 2) regression to a volume fifty percent or less than its maximum volume. Twenty-four days after treatment initiation, due to the development of tumor ulceration in some mice, all mice were sacrificed. Tumor tissue was either snap frozen for lysate preparation, frozen in optimal cutting temperature (OCT) media, or fixed in formalin.

### Flow cytometry

For *in vitro* flow cytometry experiments, cells were seeded in 6-well plates at a density of 2x10^5^ cells per well, and allowed to settle for 24 hours. Fluorescent particle was then administered. Twenty-four hours after administration, cells were washed with PBS, trypsinized, washed with PBS again, and subjected to flow cytometry.

Flow Cytometry was conducted using an LSRII cytometer (BD Biosciences, Franklin Lakes, NJ, USA). Preparation of cells in culture for single cell suspension consisted of trypsinization followed by one wash with FBS, then two washes with FACS buffer (1L 1x DPBS with 25 mM HEPES buffer, 5 mM EDTA, and 1% FBS). Gating and analysis were performed using FlowJo. Quasar705 was detected in the AF700 channel. For analysis, the cutoff for Quasar705 (AF700) positivity was chosen at the point where >99% of control cells were negative. Identical gates were then applied to all samples. Cells below the cutoff were deemed ‘NP negative’ while cells above the cutoff were considered ‘NP positive.’

### Quantitative real-time PCR

RNA was isolated from cells by suspension in Trizol (Life Technologies, Carlsbad, CA, USA), 1 mL per well for 6-well plates, followed by a Qiagen RNeasy RNA isolation kit (Hilden, Germany). RNA was quantified using a Qubit fluorometer (Thermo-Fischer Scientific, Grand Island, NY, USA). cDNA was produced from 200 ng RNA using a high-capacity cDNA reverse transcription kit (Applied Biosystems, Foster City, CA, USA). Primers (KRAS, beta-2-microglobulin, beta-actin, 18s rRNA) were obtained from Integrated DNA Technologies (Coralville, IA, USA). Real-Time PCR was performed on an Applied Biosystems 7500 Fast Real-Time PCR System (ThermoFischer Scientific, Grand Island, NY, USA) with SYBR Green reagents. PCR was done with reverse-transcribed RNA and 500 ng/μL sense and antisense primer in 20 μL reactions. 40 cycles were performed, with each cycle consisting of 15 seconds of denaturation at 95 °C followed by 1 minute of annealing and extension at 60 °C. Beta-2-microglobulin expression consistently had the lowest standard deviation among candidate housekeeping genes (beta-actin, GAPdH, 18s rRNA) and was therefore selected as the reference gene.

### Immunoblotting

Protein lysates were prepared using a Santa Cruz RIPA buffer system (Dallas, TX, USA) and quantified using a BCA Protein Assay kit (Lambda Biotech, Inc. Cat # G1002). Lysates (60 μg/lane) were loaded and separated on a 4–12% Bis-Tris gel using a Biorad Electrophoresis system (Hercules, CA, USA). Proteins were transferred onto a PVDF membrane by wet electrophoresis, blocked with 5% milk for 1 hour at 4 °C, and incubated with primary antibody [KRAS, 1:2,000, Abcam ab55391; Vinculin, 1:10,000, Abcam ab129002; Caspase 3, 1:3000, Cell Signaling 9662S; Cleaved Caspase 3, 1:1000, Cell Signaling 9661S; Phospho-p44/42 MAPK (pERK 1/2), 1:1000, Cell Signaling 9101; p44/42 MAPK (ERK 1/2), 1:1000, Cell Signaling 9102] overnight. After washing with TBST, membranes were incubated with secondary antibody [Anti-mouse IgG-HRP conjugate, 1:5,000 (KRAS), Abcam ab97023; Anti-rabbit IgG-HRP conjugate, 1:2,000 (Vinculin, Total Caspase 3, ERK1/2, phosphor ERK1/2) or 1:1,000 (Cleaved Caspase 3), Cell Signaling (7074S)] for 1 hour at 4 °C under mild agitation. Membranes were washed with TBST and incubated for 1 minute with HRP substrate (SuperSignal West Dura Extended Duration Substrate, ThermoFisher). Radiographic film was exposed to the blot and then developed in an automated radiograph developer. Various time points were used for *in vitro* studies, but for all *in vivo* data discussed mice were injected 3 times, every other day, starting after tumors reached ~0.5 centimeters, and taken down 24 hours after the final dose.

### Immunohistochemistry

Formalin fixed samples were dehydrated in sequential ethanol and then embedded in paraffin. Paraffin blocks of the samples were sectioned at 5μm thickness and then mounted to the slides. Slides were de-paraffinized and rehydrated through 2 xylene bathes, and sequential ethanol washes. Endogenous peroxidase was blocked with 0.3% H_2_O_2_ in methanol and then antigen retrieval was performed with a heated citrate buffer solution. The primary antibodies against phospho-p44/42 MAPK (Erk1/2) (Thr202/Tyr204) (Cell signaling, #4370, at 1:400) were diluted in 3% BSA in TBST and incubated at 4 degree overnight. HRP conjugated secondary antibodies was then added, washed, and finally, DAB-substrate (brown) was added for 3 minutes. This was then washed off. Counter stain was performed with Mayer’s hematoxylin (Thermo Scientific, 008011) and cover slips were mounted with Cytoseal XYL (Thermo Scientific, 8312-4). Halo Image Software Analysis (PerkinElmer) was utilized to quantify IHC signal from five 20x high powered fields (HPFs) per slide. IHC images using smooth muscle actin (SMA) and sirius red were kindly obtained from the lab of Dr. David DeNardo. [33] For *in vivo* mechanistic studies utilizing immunohistochemistry, a cohort of 7 mice per group (untreated, SC-siRNA treated, or KRAS-siRNA treated) received 8 equally-spaced injections over 2.5 weeks, followed by mouse takedown 1 week after stopping treatments.

### Statistical analysis

For animal experiments, 10 mice were assigned per treatment group. Tumor volumes for each group were compared using the Student *t* test (equal variances) or Welch’s *t* test (unequal variances) for comparisons of two groups. For comparisons among multiple groups, ANOVA was used. If the ANOVA was statistically significant, Tukey’s multiple comparison was used to compare between individual groups. All data were normal according to D’Agostino-Pearson omnibus and Kolmogorov-Smirnov testing. A *p* value ≤0.05 was deemed statistically significant. All statistical tests were two-sided and were performed using GraphPad Prism 7.01.

## SUPPLEMENTARY MATERIALS


