# Supplementary material for: Precision delivery of RAS-inhibiting siRNA to KRAS driven cancer via peptide-based nanoparticles
Source: Oncotarget. 2019 Jul 30;10(46):4761–75. doi: 10.18632/oncotarget.27109 (PMC6677667; doi:10.18632/oncotarget.27109)
Supplement: Supplementary file 1 [file oncotarget-10-4761-s001.pdf]

Precision delivery of RAS-inhibiting siRNA to KRAS driven cancer via peptide-based nanoparticles

SUPPLEMENTARY MATERIALS

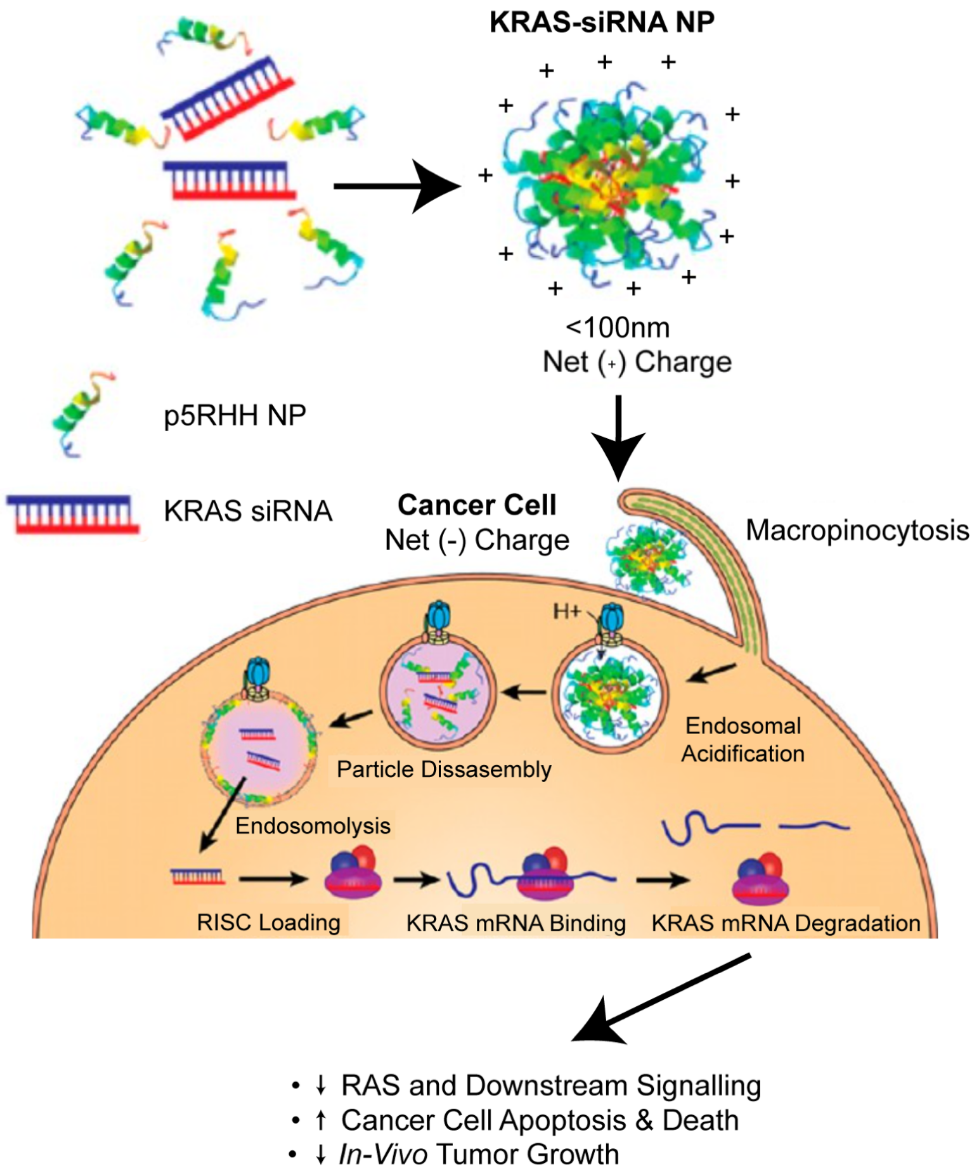

Supplementary Figure 1. p5RHH Anti-KRAS Nanoparticle Schematic. RISC= RNA induced silencing complex.

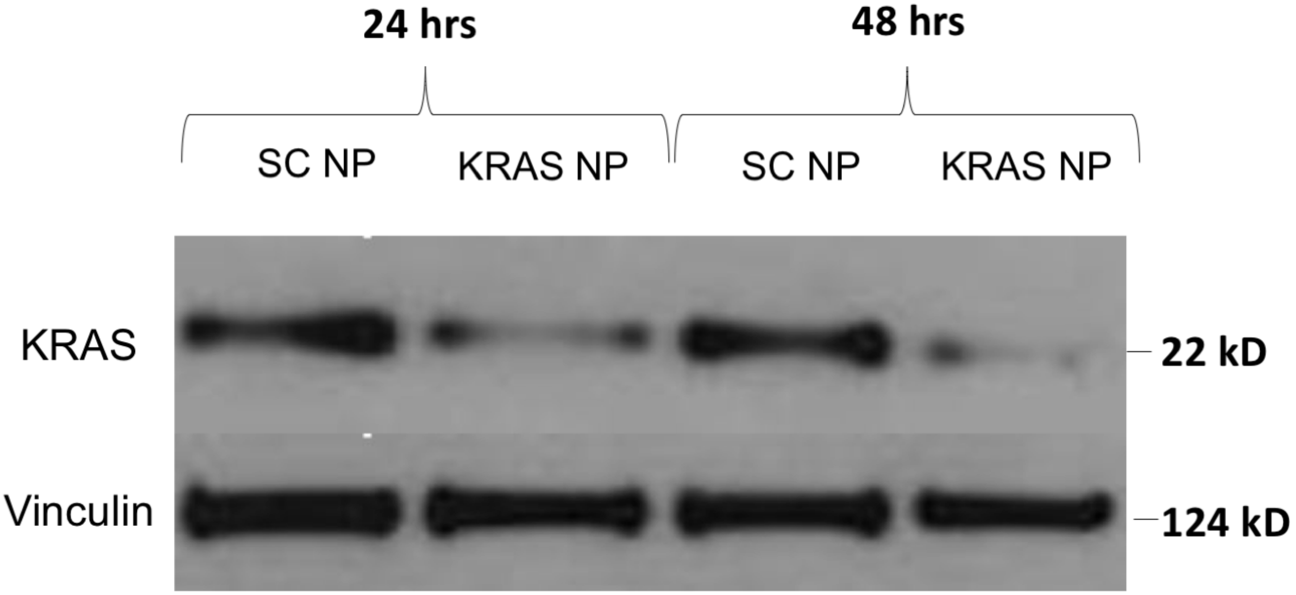

Supplementary Figure 2: Western blot demonstrating sustained KRAS knockdown *in vitro*. Western blot 24 and 48 hours after treatment of KPC-1 pancreatic cancer cells with KRAS-siRNA NP.

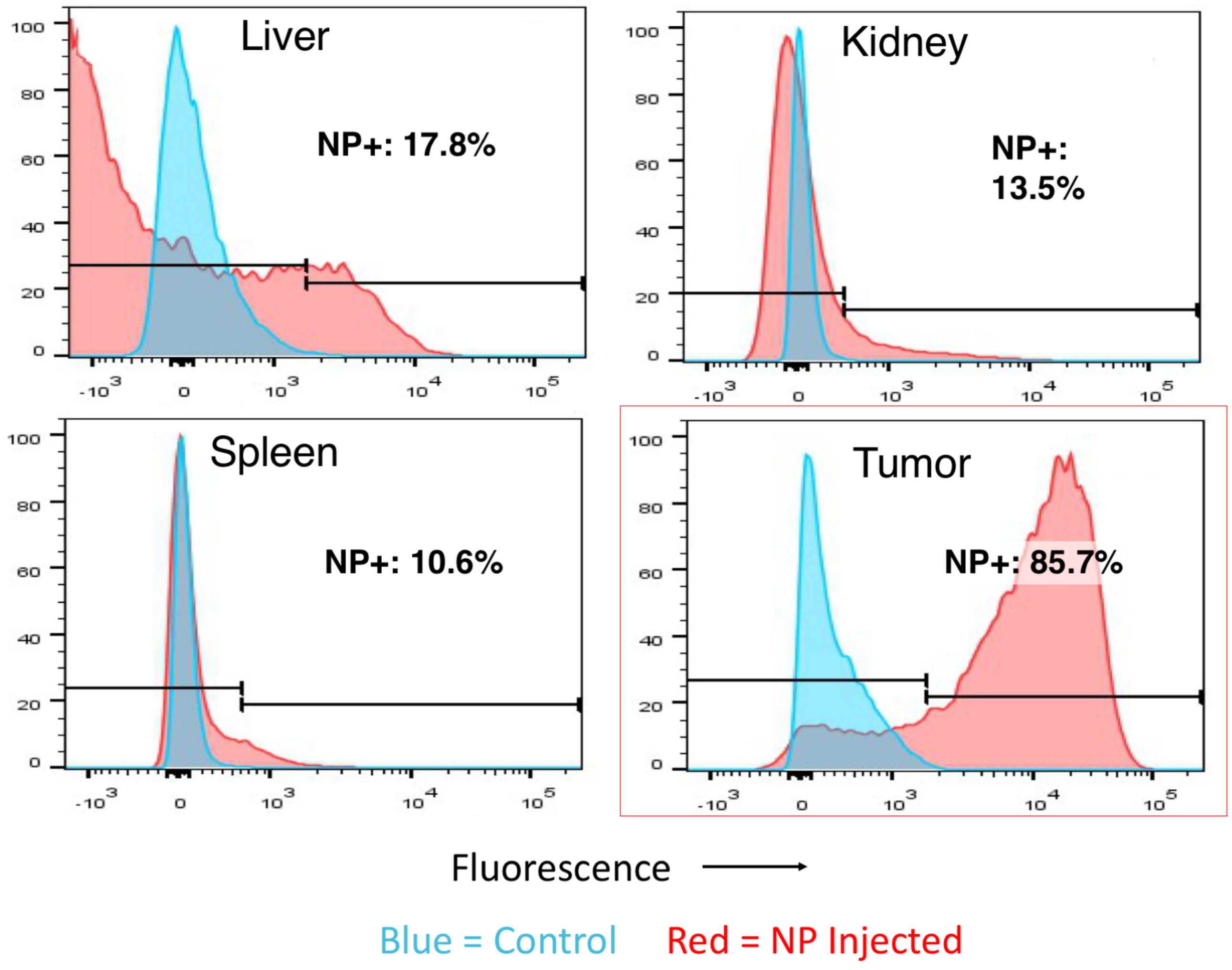

Supplementary Figure 3: Representative flow cytometry plot of single cell suspensions derived from tumor and organs of tumor-bearing mice. Flow cytometry plots after *in vivo* injection of fluorescent NP, showing avid uptake in tumor cells, but minimal uptake in other organs.

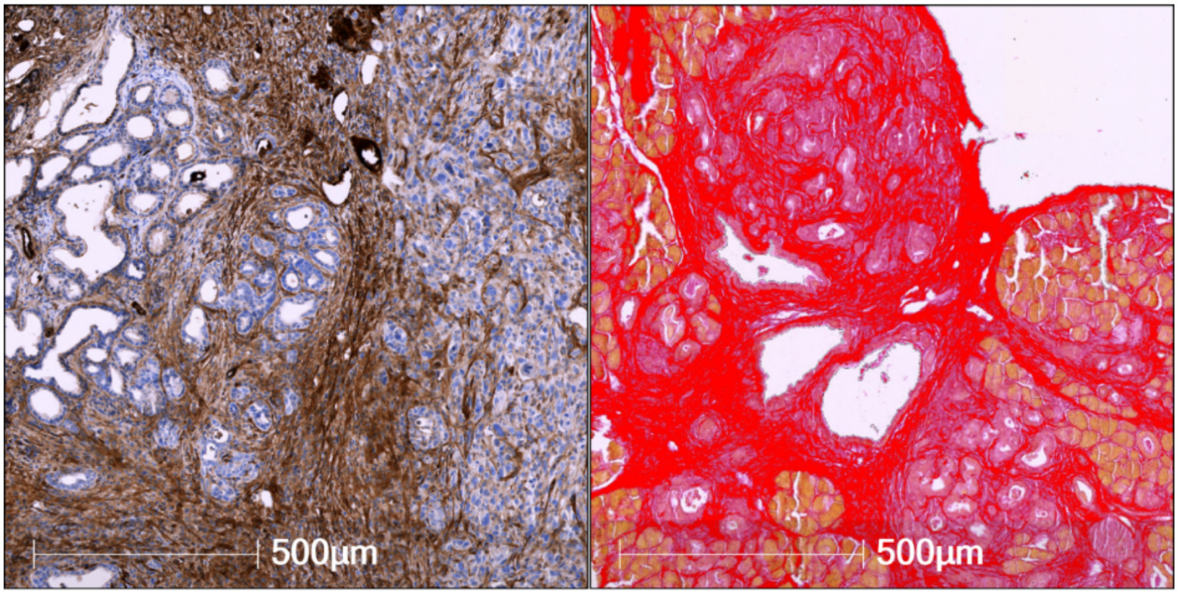

Supplementary Figure 4: KPPC tumors demonstrate a dense stromal infiltrate. Shown via SMA (left) and Sirius Red (right) staining.

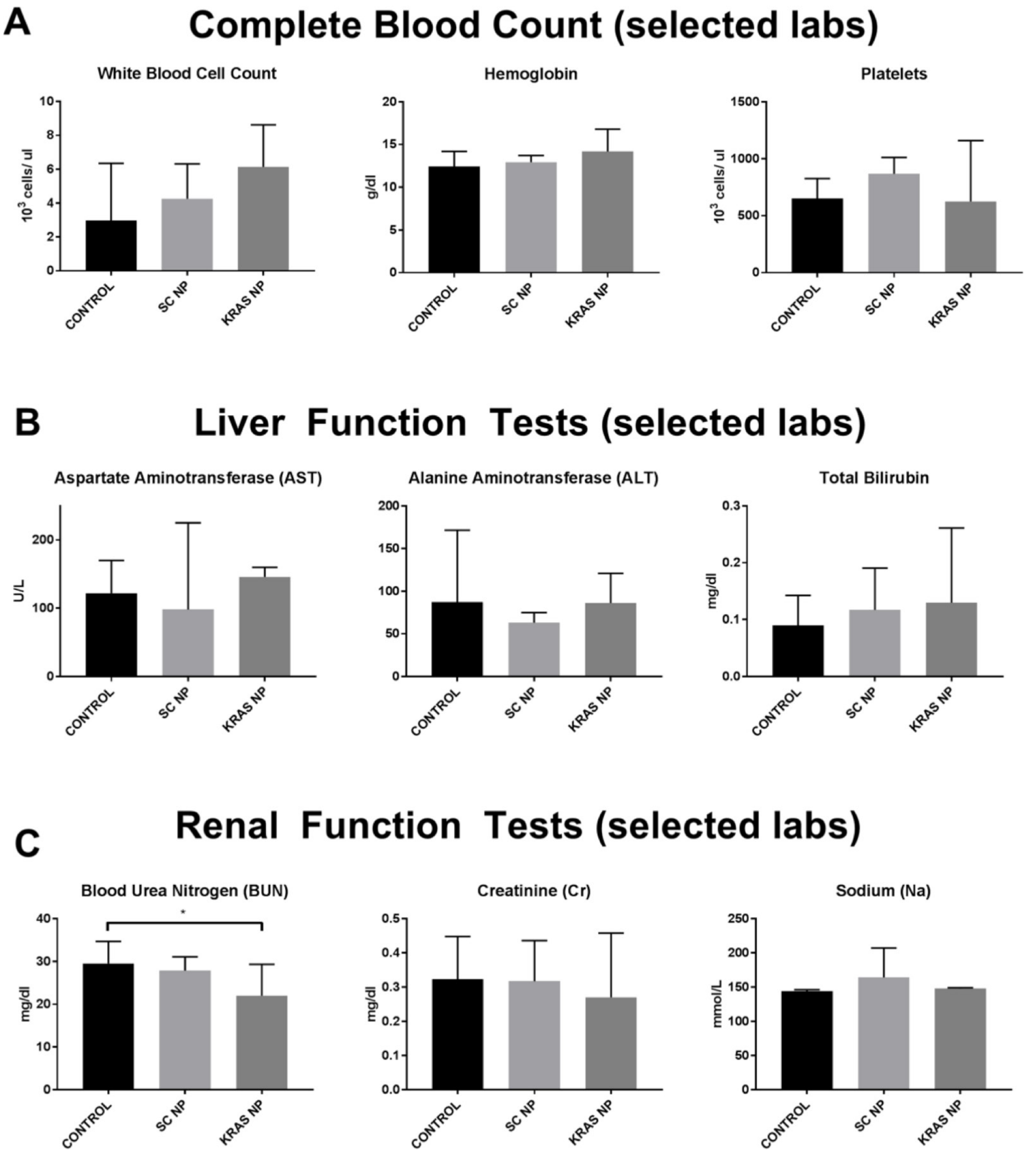

Supplementary Figure 5: Peripheral blood tests results after *in vivo* nanoparticle treatment. Results from C57BL/6J mice with ~0.5cm tumors, after 3 doses every 48 hours of control, SC-siRNA NP or KRAS-siRNA NP (N=5-8/group). Mice taken down ~6 hours after the final dose. (**A**) white blood cell (WBC), hemoglobin (Hb) and platelet counts from complete blood count. (**B**) Selected liver function test results: aspartate aminotransferase (AST), alanine aminotransferase (ALT) and total bilirubin. (**C**) Selected renal function test results: blood urea nitrogen (BUN), creatinine (Cr), and sodium (Na). $^*=p < 0.05$, 95% confidence intervals shown. Normal average lab values for similar aged female C57BL/6J mice (Jackson Laboratory data): WBC 3.48, Hb 17, platelets 1019, ALT 43, bilirubin 0.5, BUN 27, Cr 0.3, and Na 154.

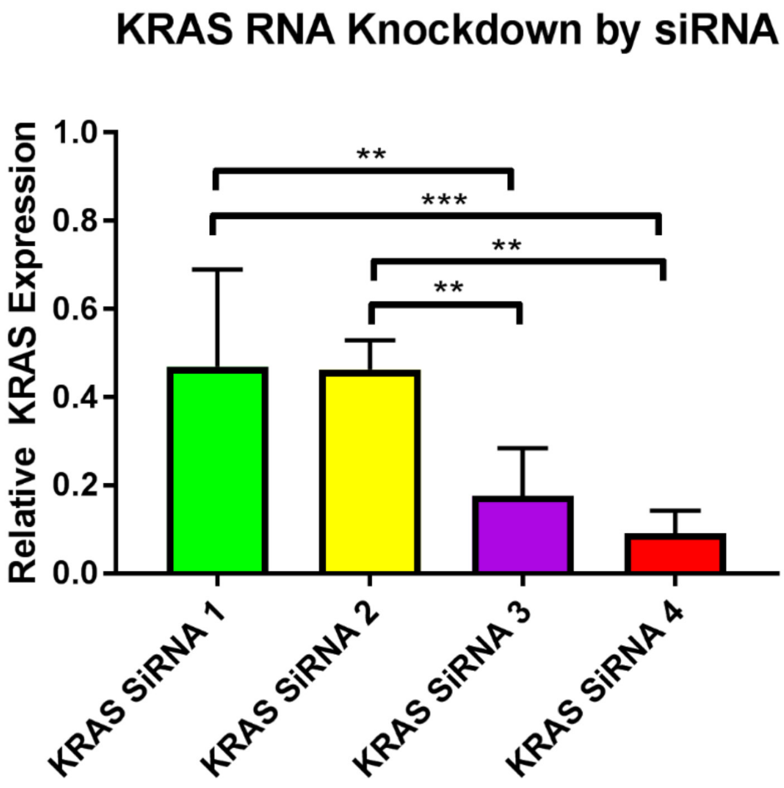

Supplementary Figure 6. RT-PCR results from an siRNA KRAS knockdown screen. Sequence 4, Sigma-Aldrich KRAS siRNA sequence GUGCAAUGAGGGACCAGUA (5'-3') was the most efficacious at KRAS knockdown *in vitro.*

Supplementary Table 1: Breakdown of positive cells per each of the five 20× HPFs for the 7 mice in the Control (A), SC-siRNA NP (B) and KRAS-siRNA NP (C) groups

A.

Control NP

415	6	1083	995	2996	1419	2915
902	4	6	1485	2937	556	2826
8	0	158	1049	2852	202	2880
0	4	830	1863	2638	255	2890
26	0	651	347	2640	1327	2952
270	3	546	1148	2813	752	2893

B.

Scramble NP

42	274	966	1456	856	131	281
2	1131	786	493	772	10	429
6	38	816	64	1200	51	133
26	36	1076	307	3	316	76
128	5	554	674	81	149	0
27	297	840	588	582	131	184

C.

KRAS NP

51	1	26	11	100	44	439
40	2	74	0	213	7	349
30	9	61	84	473	5	145
8	6	8	0	78	26	341
14	6	18	1	249	128	158
29	5	37	19	222	42	304

Each column represents a unique mouse, and each row represents one 20x HPF. Bottom row represents average number of cells positive per HPF for a given mouse.
